# Targeting Mitochondrial Damage as a Therapeutic for Ileal Crohn’s Disease

**DOI:** 10.3390/cells10061349

**Published:** 2021-05-29

**Authors:** Kibrom M. Alula, Dakota N. Jackson, Andrew D. Smith, Daniel S. Kim, Kevin Turner, Elizabeth Odstrcil, Benny A. Kaipparettu, Themistocles Dassopoulos, K. Venuprasad, Linda A. Feagins, Arianne L. Theiss

**Affiliations:** 1 Division of Gastroenterology and Hepatology, University of Colorado School of Medicine, Aurora, CO 80045, USA; Kibrom.alula@cuanschutz.edu; 2 Baylor University Medical Center, Department of Internal Medicine, Division of Gastroenterology, Baylor Scott & White Research Institute, Dallas, TX 75246, USA; dnjackson12@gmail.com (D.N.J.); elizabethodstrcil@sbcglobal.net (E.O.); themistocles.dassopoulos@bswhealth.org (T.D.); 3 Department of Medicine, Veterans Affairs North Texas Health Care System, Dallas, TX 75216, USA; andrewdavidsmith88@gmail.com (A.D.S.); Daniel.kim1@va.gov (D.S.K.); Linda.Feagins@austin.utexas.edu (L.A.F.); 4 University of Texas Southern Medical Center, Department of Internal Medicine, College of Medicine, Dallas, TX 75390, USA; kevin.turner@informdx.com (K.T.); Venuprasad.Poojary@UTSouthwestern.edu (K.V.); 5 University of Texas Southern Medical Center, Department of Immunology, College of Medicine, Dallas, TX 75390, USA; 6 Department of Molecular and Human Genetics, Baylor College of Medicine, Houston, TX 77030, USA; kaippare@bcm.edu; 7 Department of Medicine, Dell Medical School, University of Texas at Austin, Austin, TX 78712, USA

**Keywords:** inflammatory bowel diseases, antioxidant, Type I Paneth cell phenotype, epithelial cells

## Abstract

Paneth cell defects in Crohn’s disease (CD) patients (called the Type I phenotype) are associated with worse clinical outcomes. Recent studies have implicated mitochondrial dysfunction in Paneth cells as a mediator of ileitis in mice. We hypothesized that CD Paneth cells exhibit impaired mitochondrial health and that mitochondrial-targeted therapeutics may provide a novel strategy for ileal CD. Terminal ileal mucosal biopsies from adult CD and non-IBD patients were characterized for Paneth cell phenotyping and mitochondrial damage. To demonstrate the response of mitochondrial-targeted therapeutics in CD, biopsies were treated with vehicle or Mito-Tempo, a mitochondrial-targeted antioxidant, and RNA transcriptome was analyzed. During active CD inflammation, the epithelium exhibited mitochondrial damage evident in Paneth cells, goblet cells, and enterocytes. Independent of inflammation, Paneth cells in Type I CD patients exhibited mitochondrial damage. Mito-Tempo normalized the expression of interleukin (IL)-17/IL-23, lipid metabolism, and apoptotic gene signatures in CD patients to non-IBD levels. When stratified by Paneth cell phenotype, the global tissue response to Mito-Tempo in Type I patients was associated with innate immune, lipid metabolism, and G protein-coupled receptor (GPCR) gene signatures. Targeting impaired mitochondria as an underlying contributor to inflammation provides a novel treatment approach for CD.

## 1. Introduction

Crohn’s disease (CD) is a type of inflammatory bowel disease (IBD) characterized by chronic relapsing intestinal inflammation involving the ileum in ~75% of patients [1]. Although the etiology of CD is unknown, it is thought to be a multifactorial condition resulting in heterogeneous disease across patients and presenting a challenge for therapeutic strategies. Several CD susceptibility genes integrate into cellular processes that converge on the homeostasis of Paneth cells, which are long-lived cells residing at the base of the small intestinal crypts interspersed between Lgr5-expressing intestinal stem cells (ISCs). Paneth cells produce and secrete antimicrobial peptides that are essential in epithelial defense and that shape the microbiota composition. Additionally, Paneth cells produce and secret important niche factors for neighboring ISCs, thereby promoting crypt homeostasis [2]. Previous studies have demonstrated that a subset of CD patients have Paneth cell defects (called Type I Paneth cell phenotype) and proposed that Paneth cell phenotyping can stratify CD patients by disease mechanism to enhance treatment outcomes [3]. Paneth cell abnormalities in CD patients are independent of inflammation, associated with early disease recurrence after resection, gut microbial dysbiosis, and decreased mucosal expression of genes that regulate oxidative phosphorylation, the latter suggesting that these patients may have mitochondrial alterations [3,4,5,6,7,8]. A recent study from our group implicated mitochondrial dysfunction specifically in Paneth cells as a mediator of spontaneous CD-like ileitis in mice [9].

Mitochondria are multifunctional organelles involved in energy production, reactive oxygen species (ROS) production, the induction of apoptosis, and control of intracellular calcium level. Additionally, mitochondria are important cellular signaling hubs, producing signaling intermediates in response to external stimuli. In the intestine, mitochondrial metabolism and function are involved in the activation of immune cells, maintenance of the epithelial barrier, and ISC homeostasis [10,11]. A series of studies have revealed the involvement of mitochondrial stress in IBD, with altered mitochondrial health implicated by both RISK (CD) and PROTECT (ulcerative colitis) pediatric gene expression studies [12,13]. Circulating mitochondrial DNA, a pro-inflammatory mediator, is released during active disease in CD and ulcerative colitis patients and can serve as a biomarker of disease severity [14]. Additionally, induction of the mitochondrial unfolded protein response has been demonstrated in the epithelial cells of CD and ulcerative colitis patients [15]. Overall, 5% of genes highlighted by IBD genome-wide association studies are functionally linked to the maintenance of mitochondrial health [16], further underpinning the role of deregulated mitochondrial function in IBD. In this prospective study, we sought to determine mitochondrial damage in the epithelium, including Paneth cells, during active and inactive disease and the transcriptomic response to mitochondrial therapeutic targeting in ileal CD.

## 2. Materials and Methods

### 2.1. Patients

A total of 50 CD patients and 49 non-IBD controls were prospectively recruited from Baylor University Medical Center, Dallas, Texas and the Dallas Veterans Affairs Medical Center from April 2016 to August 2018. Non-IBD controls underwent an elective endoscopy for clinical purposes, did not have a history of IBD, and lacked endoscopic and histological inflammation. Biopsies were taken from the terminal ileum (from sites of endoscopically active and inactive disease if present for CD patients) using standard or jumbo forceps from the same site with the same endoscopic appearance to reduce heterogeneity across biopsies. During endoscopy, the degree of activity in the terminal ileum was scored using the Simple Endoscopic Score for Crohn’s Disease (SES-CD), a validated endoscopic scoring system for mucosal inflammation [17]. A score of 0 identified inactive ileitis, and scores ≥1 identified active ileitis [17]. Biopsies were immediately prepared in the endoscopy suite for downstream readouts: for electron microscopy, biopsies were placed in 2% glutaraldehyde solution and stored at 4 °C until being processed; for immunofluorescent staining, biopsies were embedded in OCT and immediately frozen; for ex vivo Mito-Tempo treatment, biopsies were placed in culture media on ice. To test the role of dampened mtROS signaling in active disease, only active CD biopsies were treated with Mito-Tempo and processed for global RNA sequencing (RNA-seq).

### 2.2. Paneth Cell Phenotype Analysis—Immunofluorescent Staining of Lysozyme and Quantitation of Lysozyme Granule Allocation Pattern

OCT-embedded biopsies were sectioned and incubated with lysozyme antibody (W-20, sc-27956, Santa Cruz Biotechnology, Dallas (TX), USA) at 1:75 dilution followed by secondary antibody Rhodamine Red™-X (RRX) AffiniPure Donkey Anti-Goat IgG (711-296-152, Jackson Immuno, West Grove (PA), USA) at 1:500 dilution. Sections were then stained with 4′,6-diamidino-2-phenylindole dihydrochloride (DAPI, D9542, Sigma Aldrich, St Louis (MO), USA) at 1:1000 for 5 min. For Paneth cell phenotyping classification, only samples with an adequate number of crypts (≥40) were included, as previously described [5]. Each Paneth cell was observed in a blind fashion for disease status for lysozyme granule allocation pattern as previously described [3,4,5,6,18,19]. Briefly, each Paneth cell was classified into the following categories: normal, disordered (abnormal distribution and size of granules), diminished (≤10 granules), diffuse (no recognizable granules), excluded (majority of granules do not contain lysozyme stain), or enlarged (lower number of megagranules) [19]. The percentage of abnormal Paneth cells within each sample was calculated to define the Paneth cell phenotype as previously described; the Type I Paneth cell phenotype was defined as ≥20% of total Paneth cells demonstrating abnormal lysozyme granule allocation patterns and the Type II Paneth cell phenotype was defined as <20% of total Paneth cells demonstrating abnormal lysozyme granule allocation patterns [3,4,5,6,18,19].

### 2.3. Transmission Electron Microscopy (TEM)

Biopsies were fixed in 2% glutaraldehyde in 1× PBS, dehydrated and embedded in epoxy resin for electron microscopy. Ultrathin 70 nm sections were examined on a transmission electron microscope (TEM) (Hitachi BioMedical, Twinsburg, OH, USA). For each patient, we observed mitochondria in 3 different cell types: Paneth cells, goblet cells, and enterocytes. Each mitochondrion was observed in a blind fashion and classified as having normal or abnormal ultrastructural features (altered morphological features including dissolved cristae and electron-dense inclusion bodies). Per patient, an average of 320 mitochondria in Paneth cells were quantitated (20 ± 0.5 Paneth cells per patient with an average of 16 mitochondria per Paneth cell). An average of 300 mitochondria in goblet cells were quantitated (20 goblet cells per patient with an average of 15 ± 0.8 mitochondria per goblet cell) and an average of 1520 mitochondria in enterocytes were quantitated (40 enterocytes per patient with an average of 38 ± 0.6 mitochondria per enterocyte). In Paneth cells, individual mitochondria were analyzed for the following morphological features: perimeter (nm) by manual tracing in ImageJ software, circularity (4π(area/perimeter^2^), number of cristae, and number of electron-dense inclusion bodies.

### 2.4. CD Risk Allele Analysis

The presence of single nucleotide polymorphisms (SNPs) for *ATG16L1* (rs2241880), and *NOD2* (rs2066844, rs2066845, rs2066847) was identified during RNA-seq analysis (Novogene, Beijing, China) that involved alignment (picard tools v1.111 and samtools v0.1.18), calling SNP (HaplotypeCaller GATK4.1 version), and SNP annotation (annovar) against dbSNP and other databases.

For patients who were only able to provide inactive disease biopsies (which were excluded from RNA-seq analysis since only active biopsies were analyzed), *ATG16L1* (rs2241880) was genotyped by Sanger sequencing. Genomic DNA was isolated from patient biopsies using AllPrep DNA/RNA kit (69504, Qiagen, Hilden, Germany). Genomic DNA for *ATG16L1* was amplified by PCR with Platinum Taq DNA Polymerase High Fidelity (11304-011, Invitrogen, Carlsbad, CA, USA), dNTP Mix, PCR Grade (201900, Qiagen), and the following primers from Invitrogen: forward: 5′-ACAGGTTAGTGTGCAGGAGA-3′ and reverse: 5′-CAGTCAGCTCTGCCATTACA-3. PCR conditions: 95 °C 2 min, 33 cycles, 95 °C 30 s, 53 °C 20 s, 72 °C 30 s. The amplified product was purified using QIAquick PCR Purification Kit (28104, Qiagen). Sanger sequencing was conducted by Eurofins using the following sequencing primer: 5′-TGCAGGAGAGTAAGGCATGT-3′.

### 2.5. Ex Vivo Mito-Tempo Treatment

Biopsies obtained from active sites of CD and non-IBD control biopsies were placed in 300 μL DMEM culture media in the endoscopy suite and placed on ice. Within 30 min, the biopsies were transported to the lab, dabbed to remove excess culture media, and weighed. Biopsies were then incubated in 300 μL DMEM culture media containing 40 mg/L penicillin, 90 mg/L streptomycin, and 0.5 μg/g tissue Mito-Tempo or vehicle; two biopsies from each patient were used, one biopsy treated with Mito-Tempo and one with vehicle. Biopsies were cultured in a humidified environment at 37 °C and 5% CO_2_ for 2 h then moved from the culture media and incubated in RNAlater (Qiagen).

### 2.6. Global RNA Sequencing (RNA-Seq)

**Figure 1:**
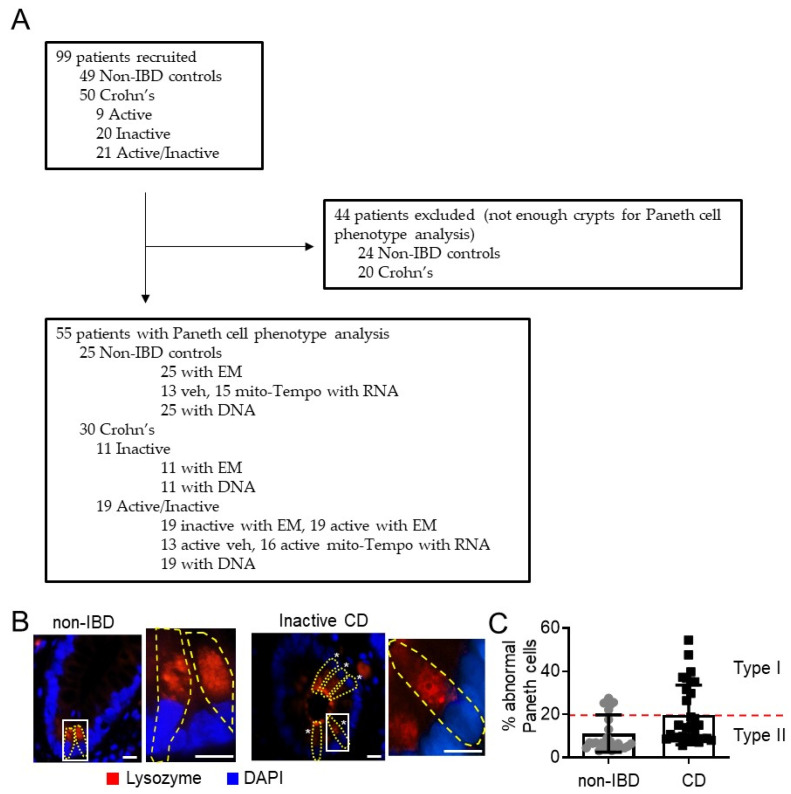
Flowchart of recruited and excluded patients and Type I Paneth cell phenotyping. (**A**) Number of patients recruited into analysis for Paneth cell phenotyping, EM, RNA-seq, and presence of autophagy-related SNPs. (**B**) Example of lysozyme immunofluorescent staining for Paneth cell (yellow outline) phenotype analysis. Star denotes abnormal lysozyme allocation pattern. Box denotes area of higher magnification. Scale bars: 10 μm. (**C**) % abnormal Paneth cells. A minimum of 40 well-oriented crypts were quantitated for each patient.

Total RNA was isolated from patient biopsies using AllPrep DNA/RNA kit (69504, Qiagen). Samples were sent to Novogene for RNA sequencing. Of the active biopsies from 19 CD patients, 16 treated with Mito-Tempo and 13 treated with vehicle had transcriptome data that passed quality control and library construction (Figure 1). Of the remaining 25 non-IBD patients, 15 biopsies treated with Mito-Tempo and 13 biopsies treated with vehicle had transcriptome data that passed quality control and library construction. Alignments were parsed using the STAR program and differential expressions were determined through DESeq2/edgeR. GO and KEGG enrichment were implemented by the ClusterProfiler. Reference genome and gene model annotation files were downloaded from genome website browser (NCBI/UCSC/Ensembl) directly. Indexes of the reference genome was built using STAR and paired-end clean reads were aligned to the reference genome using STAR (v2.6.1). STAR used the method of maximal mappable prefix (MMP), which can generate a precise mapping result for junction reads. STAR counted the number of reads per gene while mapping. The counts coincide with those produced by htseq-count with default parameters. Then, the FPKM of each gene was calculated based on the length of the gene and reads count mapped to this gene. Differential expression analysis between two groups was performed using the DESeq2 R package (1.14.1). IDEP.91, a web-based program (http://bioinformatics.sdstate.edu/idep/, accessed on 15 March 2021) was used to further analyze and identify pathways related to up- and downregulated gene expression as a result of Mito-TEMPO treatment of CD patients by Reactome enrichment pathway analysis of differentially expressed genes. Heatmaps were generated using the web-based program called Heatmapper (http://www.heatmapper.ca/expression/, accessed on 15 March 2021).

### 2.7. Quantitative Real-Time PCR Analysis

For graphical representation of quantitative PCR data, the ∆∆C_T_ was calculated as follows: ∆∆C_T_ = (Ct, _target_ − Ct, _18S_) *_all other treatment groups_* − (Ct, _target_ − Ct, _18S_) _non-IBD veh_, with the final graphical data derived from 2^−∆∆CT^. Primers from Integrated DNA Technologies: FAS forward: 5′-GTGATGAAGGACATGGCTTAGA-3′ and FAS reverse: 5′-GGGTCACAGTGTTCACATACA-3. ACADS forward: 5′-CATCTACCAGTCTGTGGAACTG-3′ and ACADS reverse: 5′-GCTGGGAAGAGATGTTCCTTAT-3. FAAH forward: 5′-GGTGCAGAAGTTACACAGTAGAG-3′ and FAAH reverse: 5′-AGTCTCACAGTCAGCCAGATA-3. IL17F forward: 5′-TGTGCCAGGAGGTAGTATGA-3′ and IL17F reverse: 5′-GGTCCCAAGTGACAGTGTAAT-3. IL23A forward: 5′-GGGACACATGGATCTAAGAGAAG-3′ and IL23A reverse: 5′-TGCAAGCAGAACTGACTGT-3. 18S forward: 5′-CCCCTCGATGCTCTTAGCTGAGTG-3′ and 18S reverse: 5′-CGCCGGTCCAAGAATTTCACCTCT-3.

### 2.8. Statistics

Data represent individual data points ± SEM. For patient demographics, a non-parametric Mann–Whitney–Wilcoxon test was used for continuous variables and a chi-square/Fisher’s exact test was used for associations between characteristics. Spearman rank was used to calculate the association between % of abnormal Paneth cells and % of Paneth cells with unhealthy mitochondria. An unpaired two-tailed *t* test was performed for the comparison of means between two groups. Multiple comparisons were conducted with either one-way or two-way ANOVA followed by a Bonferroni post hoc test (GraphPad Prism 8.4.2, San Diego, CA, USA). For transcriptome analysis, differential expression analysis between two groups was performed using the DESeq2 R package (1.14.1). The resulting *p*-values were adjusted using Benjamini and Hochberg’s approach for controlling the false discovery rate (FDR). Genes with an adjusted *p*-value < 0.05 found by DESeq2 were assigned as differentially expressed. Reactome terms with a *p*-value < 0.05 were considered significantly enriched by differential expressed genes.

## 3. Results

### 3.1. Mitochondria Exhibit Damage in Paneth Cells, Goblet Cells, and Enterocytes during Active CD and in Paneth Cells of Type I CD Patients Independent of Inflammation

A novel cohort of patients was recruited from two centers in Dallas, TX to investigate mitochondrial health in Paneth cells of the terminal ileum (Table 1). Non-IBD control subjects were significantly older than CD patients (*p* = 0.0002, Mann–Whitney–Wilcoxon test). No differences were noted between CD and non-IBD patients with regard to gender, race, reported smoking status, or co-morbidities. After Paneth cell phenotype analysis, 20 CD patients and 24 non-IBD patients were excluded as the biopsy samples had an inadequate number of crypts (<40 artifact-free crypts [5]) for Paneth cell phenotyping (Figure 1A). The prevalence of the Type I Paneth cell phenotype in our cohort was 33% (10 of 30) for CD patients and 24% (6 of 25) for non-IBD patients (Figure 1B,C), which agrees with previous studies demonstrating that the Type I Paneth cell phenotype occurs in ~25% of adult CD patients and is independent of active inflammation [5]. Since CD genetic susceptibility variants in ATG16L1 T300A or NOD2 genes are associated with Paneth cell defects in other cohorts of patients [3,4], we next determine whether these genetic variants are present in our CD cohort. The ATG16L1 T300A variant was found in 63% (19 of 30) of CD patients and correlated with the Type I Paneth cell phenotype (*p* = 0.048, chi-square/Fisher’s exact test) as previously published. The NOD2 risk SNPs were not present in this cohort of CD patients.

**Figure 2:**
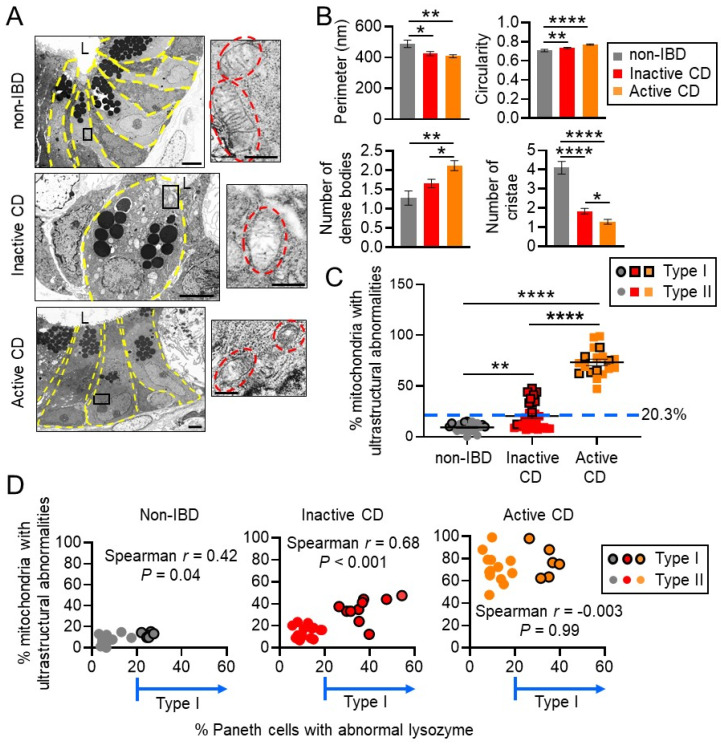
Ileal CD patients with unhealthy mitochondria stratify as Type I abnormal Paneth cell phenotype. (**A**) Representative TEM image of Paneth cell (yellow outline) mitochondria (red outline). Scale bars: 4 μm, boxed pullout: 500 nm. L, lumen. (**B**) Quantitation of mitochondrial parameters in Paneth cells measured by ImageJ fromTEM images. (**C**) Quantitation of % Paneth cell mitochondria per patient with ultrastructural abnormalities (dissolved cristae, electron-dense inclusion bodies). (**D**) Spearman’s rank correlation. Results are presented as individual data points ± SEM of 25 non-IBD and 30 inactive CD patients. * *p* < 0.05, ** *p* < 0.01, **** *p* < 0.001 by one-way ANOVA and Tukey’s post hoc test.

Specimens were processed for transmission electron microscopy (TEM) to visualize mitochondrial morphology. Quantitative measurements of mitochondria demonstrated a decreased perimeter and number of cristae and increased circularity and number of dense bodies in CD Paneth cell mitochondria compared to non-IBD controls, with the most severe changes in active CD (Figure 2A,B). In Paneth cells, ultrastructural abnormalities in mitochondria (dissolved cristae, electron-dense inclusion bodies) were demonstrated in an average of 9.2% of mitochondria of non-IBD patients, 20.3% of mitochondria of inactive CD patients, and 73.3% of mitochondria of active CD patients (Figure 2C). These results suggest that CD biopsies taken from sites of active disease exhibited a high proportion of Paneth cells with mitochondrial abnormalities, likely due to inflammatory damage, as has been shown previously [11]. Interestingly, inactive CD Paneth cells exhibited a range of mitochondrial ultrastructural abnormalities, with some patients exhibiting a normal mitochondrial pool reflecting that of non-IBD, and other patients exhibiting a larger portion of mitochondria with abnormalities (Figure 2C). The highest percentage of Paneth cells with mitochondrial abnormalities stratified as Type I Paneth cell phenotype in inactive CD and non-IBD biopsies (Figure 2D). This did not correlate in active CD biopsies, likely due to inflammation as a confounding factor (Figure 2D). These results suggest that Paneth cell defects are associated with mitochondrial ultrastructural alterations independent of inflammation.

**Figure 3:**
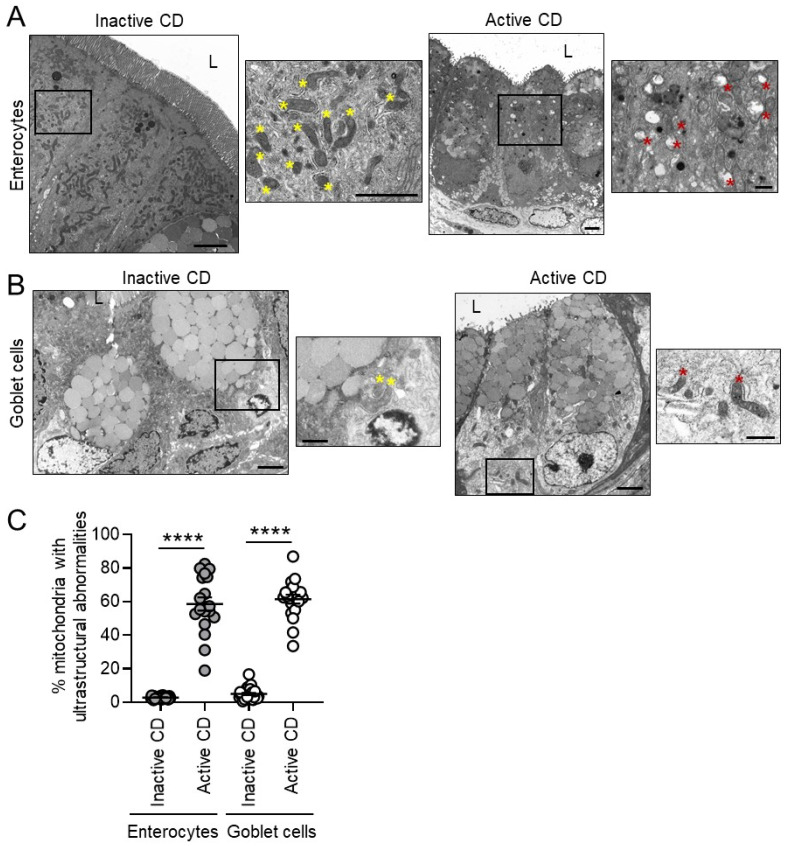
Active CD is associated with mitochondrial ultrastructural abnormalities in enterocytes and goblet cells. (**A**,**B**) Representative TEM image of enterocytes (**A**) and goblet cells (**B**). Yellow star denotes normal mitochondria, red star mitochondria with ultrastructural abnormality. Scale bars: 2 μm, boxed pullout: 1 μm. (**C**) Quantitation of mitochondria with ultrastructural abnormalities. Results are presented as individual data points ± SEM of 30 inactive and 19 active CD patients. **** *p* < 0.001 by one-way ANOVA and Bonferroni post hoc test (**C**).

Further examination of other epithelial cell types including enterocytes and goblet cells revealed increased mitochondrial ultrastructural abnormalities during active CD in these cell types (Figure 3). In inactive CD sites, enterocytes and goblet cells exhibited a low proportion of mitochondrial abnormalities in contrast to Paneth cells. Those inactive CD patients with the percentage of abnormal mitochondria above the mean (20.3%; Figure 2C) will be referred to as the abnormal Paneth cell mitochondrial phenotype herein. Of the 30 CD patients, 11 fit the classification of abnormal PC mitochondrial phenotype, which was significantly associated with the Type I Paneth cell phenotype (*p* < 0.0001, chi-square/Fisher’s exact test; Table 2). No differences were noted between the normal and abnormal Paneth cell mitochondrial phenotypes in regard to gender, age, race, disease severity (Harvey Bradshaw Index Score or the presence of endoscopic active inflammation), treatment at biopsy, smoking status, or co-morbidities (Table 2).

### 3.2. Mito-Tempo, a Mitochondrial-Targeted Therapeutic, Restores Altered CD Genes Involved in Immune Response, Apoptotic, and Metabolism Pathways to Non-IBD Expression Levels

Given emerging studies suggesting mitochondrial dysfunction in IBD [9,20] and our results demonstrating that CD biopsies taken from sites of active disease exhibited a high proportion of Paneth cells, goblet cells, and enterocytes with mitochondrial abnormalities, targeting impaired mitochondria as an underlying contributor to inflammation could provide a novel treatment approach for CD. Increased ROS derived from damaged mitochondria are a key source of cellular and tissue damage [21]. Mito-Tempo is a mitochondrial-targeted superoxide dismutase 2 (SOD2) mimetic shown to have antioxidant properties and to ameliorate ileitis in mice driven by mitochondrial dysfunction [9,22]. To demonstrate the response of mitochondrial-targeted therapeutics in CD, transcriptomic analysis of active CD biopsies treated with Mito-Tempo is an unbiased approach to assess inflammatory and other pathological pathways. Active CD biopsies and non-IBD control biopsies were incubated for 2 h in culture media containing 0.5 μg/g tissue Mito-Tempo or vehicle. This time point was chosen since it is prior to epithelial cell death [23] but allows ample transcriptomic changes which were measured by global RNA sequencing (RNA-seq).

**Figure 4:**
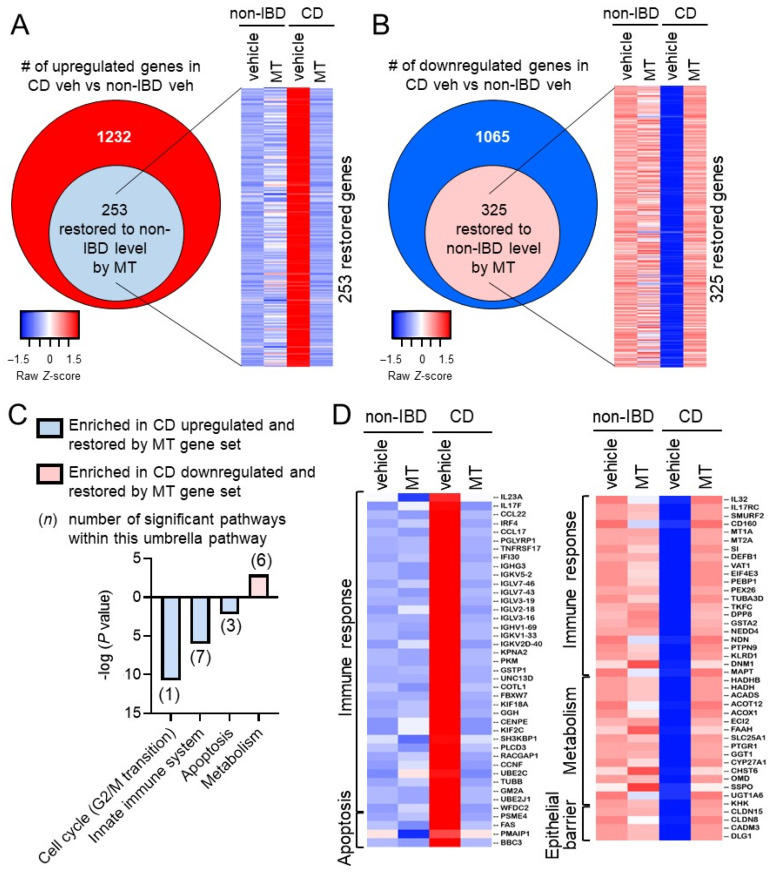
Mito-Tempo alters inflammatory response, metabolism, apoptotic, and epithelial barrier function gene signatures in CD patients. Ileal biopsies from CD and non-IBD patients were analyzed by RNA-seq after 2 h ex vivo incubation with Mito-Tempo or vehicle. (**A**,**B**) Diagrams summarizing the number of differentially expressed genes up- or down-regulated in CD and restored by Mito-Tempo treatment to non-IBD level. *p* adj < 0.05. (**C**) Significantly enriched pathways by Reactome analysis of DEGs in CD restored to non-IBD level by Mito-Tempo. (**D**) Heat maps of significantly upregulated or downregulated genes in CD restored to non-IBD expression by Mito-Tempo. *n* = 13 veh non-IBD, 15 Mito-Tempo non-IBD, 13 veh active CD, 16 Mito-Tempo active CD. *p* < 0.05 was considered significant in pathway selection.

Compared to non-IBD controls (non-IBD vehicle), there were 1232 upregulated differentially expressed genes (DEGs) and 1065 downregulated DEGs in CD patients (CD vehicle) (Figure 4A,B, Table S1). These altered DEGs in CD patients associated with gene signature pathways including metabolism, signal transduction, autophagy, and antimicrobial peptides (Figure S1). Of the 1232 upregulated genes in CD patients, 253 were restored to the non-IBD expression level by Mito-Tempo (Figure 4A and Table S2). Of the 1065 downregulated genes in CD patients, 325 were restored to the non-IBD expression level by Mito-Tempo (Figure 4B and Table S3). Therefore, Mito-Tempo normalized 25% of altered genes in active CD biopsies after only 2 h. The effect of Mito-Tempo on these genes in non-IBD patients (non-IBD Mito-Tempo vs. non-IBD vehicle) was minor compared to its effect in CD patients (Figure 4A,B). The Mito-Tempo-restored genes in CD included signatures associated with the cell cycle (specifically mitosis), innate immune system, apoptotic, and metabolism pathways (Figure 4C). Numerous genes in the interleukin (IL)-17/IL-23 signaling pathway were altered in CD biopsies and normalized by Mito-Tempo treatment (IL23A, IL17F, IL32, IL17RC, CCL22, CCL17, IRF4, PGLYRP1) (Figure 4D). The IL-17/IL-23 pathway plays a central role in mediating intestinal inflammation by controlling activation of Th1 and Th17 cells and dampening Treg cell expansion [24]. Additionally, in CD biopsies, Mito-Tempo decreased expression of antigen processing genes (IF130, TNFRSF17, IGHG3, IGKVs, IGLVs, IGHVs) and pro-apoptotic genes (FAS, PMAIP1, BBC3) (Figure 4D). Multiple immune response genes that are downregulated in CD and restored by Mito-Tempo are protective against intestinal inflammation (SMURF2, CD160, MT1A, MT2A, SI, DEFB1) [25,26,27,28] and promote epithelial barrier function (CLDN8, CLDN15, DLG1) (Figure 4D) [29,30,31]. Additionally, three of the six Reactome pathways under “Metabolism” (Figure 4C) specifically involved lipid metabolism with multiple lipid metabolism genes downregulated in CD, as previously reported [32], and normalized by Mito-Tempo treatment (HADHB, HADH, ACADS, ACOT12, ACOX1, ECI2, FAAH). Relative change in the expression of representative genes FAS (apoptosis), FAAH, ACADS (lipid metabolism), Il23A, Il17F, was similar to expression patterns demonstrated by RNAseq analysis (Figure S2). Mitochondrial biogenesis gene PPARGC1A was upregulated during CD and not significantly altered by Mito-Tempo (Figure S2). Collectively, these results suggest that Mito-Tempo exhibits therapeutic potential to normalize the expression of these CD disease pathways to levels demonstrated in non-IBD patients.

### 3.3. Paneth Cell Phenotype Is Associated with Unique Mito-Tempo-Induced Gene Signatures

**Figure 5:**
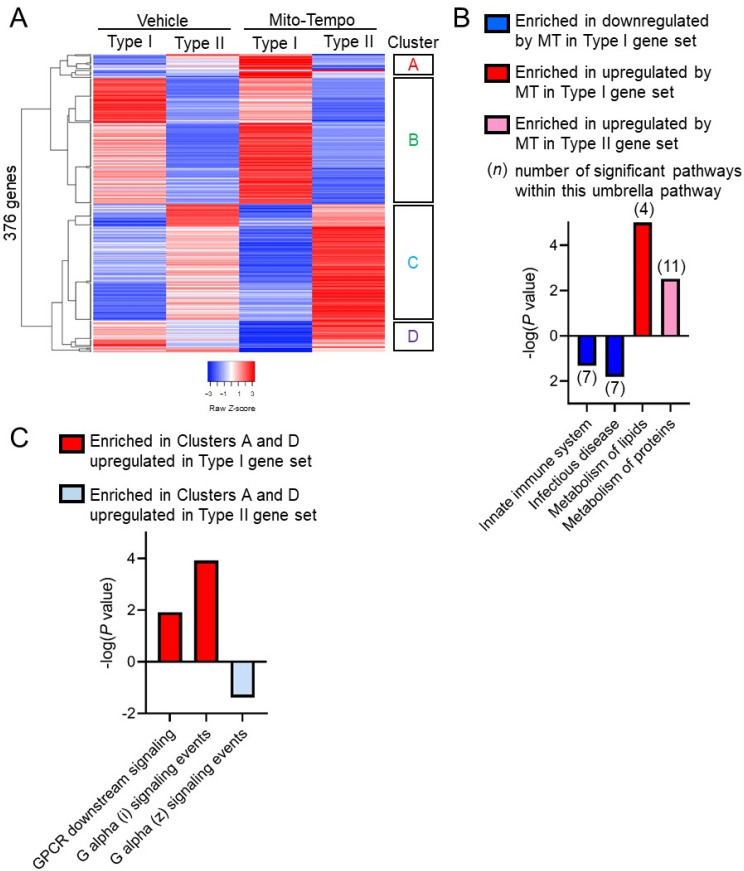
Paneth cell phenotype is associated with unique Mito-Tempo-induced gene signatures. (**A**) Heat maps based on hierarchical clustering of gene expression in Type I and Type II Paneth cell phenotype CD patients; (**B**) significantly enriched pathways by Reactome analysis of logFoldChange > 1.5 DEGs by Mito-Tempo in Type I or II patients; (**C**) significantly enriched pathways by Reactome analysis of genes in Clusters A and D shown in (**A**). *p* < 0.05 was considered significant in pathway selection.

Although Mito-Tempo likely affects numerous cell types in the whole tissue, global response to Mito-Tempo may be associated with the Paneth cell subtype of CD. Transcriptomics of Mito-Tempo treatment was stratified based on CD patient Paneth cell phenotyping as Type I or Type II. 376 genes were differentially expressed between Type I and Type II CD specimens (*p* adj. < 0.06, *p* < 0.05; Figure 5A; Table S4). Mito-Tempo-altered genes in Type I CD associated with innate immune response (SIGLEC14, SIGLEC5, DEFB124, FCN3, CHI3L1, IGHV1-69, IGHV3-7, IGHV4-34, IGHV10-54, IGHG3) and lipid metabolism pathways (RPL10L, HMGCLL1, CIDEA, GNG10, HAPLN1, DIO3, OGN, CHST13) (Figure 5B). Mito-Tempo altered genes in Type II CD associated with metabolism of proteins (GALNT13, FAP, HIST1H2BI, GP2, IGF2, PROZ, GZMB, LYPD1, ESR1, GLIS1, HIST1H4C). Genes with obvious changes by Mito-Tempo in Type I or Type II patients (clusters A and D in Figure 5A; Table S5) were also analyzed for pathway enrichment. Genes in clusters A or D were associated with significantly upregulated signaling by G protein-coupled receptors (GPCR) in Type I patients (OR52D1, RASGRF2, RGS17, ADCY4, AGTR1, RGS8, GNG10) and downregulated GPCR signaling in Type II patients (RASGRF2, RGS17, and ADCY4) (Figure 5C).

## 4. Discussion

Emerging studies linking mitochondrial health and IBD suggest the potential for mitochondrial-targeted interventions for ulcerative colitis and CD. Here, our results demonstrate that during active CD inflammation, the epithelium exhibited severe mitochondrial damage evident in Paneth cells, goblet cells, and enterocytes. Independent of inflammation, CD Paneth cells, but not goblet cells or enterocytes, exhibited mitochondrial ultrastructural abnormalities with highest proportion in patients stratifying as the Type I phenotype. Targeting impaired mitochondria as an underlying contributor to inflammation provides a novel treatment approach for CD.

Multiple mitochondrial-targeted therapeutics have been tested in preclinical models of intestinal inflammation. The majority thus far have targeted the elimination of mtROS via antioxidant function since mtROS are elevated during impaired mitochondrial function, induce cellular damage, and act as stress signaling intermediates [33]. Synthetic mtROS scavengers (Mito-Tempo and MitoQ) have been shown to ameliorate intestinal inflammation in mice and enhance barrier function in CD patient colonic biopsies [9,34,35,36]. As an alternate to targeting mtROS, a recent study demonstrated that altering mitochondrial metabolism to favor oxidative phosphorylation by treatment with dichloroacetate improved ISC function in organoids cultured from inflamed TNF^ΔARE^ mice [20]. Additionally, preclinical models have shown protection against colitis by a key regulator of mitochondrial biogenesis, PGC1α [37], but this has not been studied in CD or mouse models of ileitis. Our results demonstrate that PPARGC1A was upregulated in CD biopsies, which may represent an adaptive response to ongoing mitochondrial damage during CD inflammation and deserves further study. By means of transcriptomics, we show that Mito-Tempo rapidly normalized the expression of 25% of altered CD genes to levels demonstrated in non-IBD patients. Reactome pathway analysis revealed that the Mito-Tempo-restored genes in CD patients included signatures associated with antigen processing, lipid metabolism, apoptosis, and IL-17/IL-23 signaling, all of which are relevant to CD pathology.

Numerous antigen processing genes were altered by Mito-Tempo in CD patients. Antigen processing is an important step prior to presentation by antigen presenting cells, which include dendritic cells, macrophages, B cells, and epithelial cells in the gut. It has been demonstrated that both T cells and dendritic cells exhibit increased intracellular ROS upon antigen-specific interaction and that alteration of oxidation state interferes with bi-directional dendritic cell/T cell communication [38,39]. Specifically, ROS has been shown to be important in effective antigen cross presentation by gut dendritic cells to elicit antigen-specific T cell responses [40]. In terms of IBD, activated dendritic and T cells accumulate at sites of inflammation and targeting their activation provides a potential therapeutic target [41,42]. Our results suggest that Mito-Tempo-induced alteration of ROS influences antigen processing genes in CD patients. Further studies are needed to determine which antigen processing cell(s) are affected by ROS inhibition and how this relates to pathology in CD.

It is not surprising that lipid metabolism and apoptosis are altered by mitochondrial therapeutic targeting given that these pathways are associated with this organelle [11,33]. Redox status and the production of ROS are linked to energy metabolism, including lipid metabolism, with regulators of metabolic flux sensitive to oxidants [43]. Furthermore, during elevated ROS, lipids, especially polyunsaturated fatty acids, can be damaged by oxidants. This is called lipid peroxidation and has been shown to induce apoptosis [44]. In terms of apoptotic genes, Mito-Tempo was shown to alter gene expression of *FAS*, and BH3-only proteins *PMAIP1* (*NOXA*) and *BBC3* (*PUMA*), all of which play a critical role in the induction of cell death, dependent on oxidative stress and other cell death signals [45,46,47].

The effect of Mito-Tempo on IL-17/IL-23 signaling suggests a novel mechanism whereby mtROS modulates CD inflammation via IL-17/IL-23, the targeting of which by mirikizumab or guselkumab was recently reported to have benefits in phase 2 clinical trials for CD [44,48]. The role of mitochondrial-derived ROS has been shown to be crucial for CD4^+^ T cell activation and differentiation [49,50]. However, the mechanism whereby mitochondrial-derived ROS exhibits this effect on T cells is not fully elucidated and could involve direct ROS signaling and/or ROS influence of the redox state of the cell. Mitochondrial-derived ROS-mediated activation of pathogenic Th17 cells producing IL-17 and IL-23 has not been previously studied in IBD patients. However, studies of CD4^+^ T cells in rheumatoid arthritis patients demonstrated that differentiation to pathogenic Th17 cells was dependent on increased ROS production, which could be inhibited by Mito-Tempo [51]. Our results corroborate these findings with decreased IL23A, IL17F, IL32, and IL17RC expression in CD biopsies treated with Mito-Tempo. IL-17F and IL-17A are produced by type 3 innate immune cells (ILCs) and subsets of T cells including Th17 cells, CD8^+^ T cells, natural killer T cells, and γδ T cells [52]. IL-17F, but not IL-17A, is secreted by activated monocytes, basophils, mast cells, and gut epithelial cells and has greater affinity for IL-17RC, which is dominantly expressed in non-hematopoietic cells.

Whether Paneth cell defects in humans cause mitochondrial dysfunction or vice versa remains unknown. Our recent study in mice demonstrated that the induction of mitochondrial dysfunction by deletion of *Phb1* in intestinal epithelial cells triggered Paneth cell abnormalities and spontaneous ileitis in mice [9]. Paneth cell-specific deletion of *Phb1* driven by *Defα6-Cre* or *Mist1-CreER^T2^* was sufficient to drive spontaneous ileitis, suggesting a causative role of mitochondrial dysfunction in ileitis that initiates in Paneth cells [9]. Additionally, induction of mitochondrial stress specifically in *Lgr5+* ISC via *Hsp60* deletion causes the development of abnormal Paneth cells [20]. These results suggest that mitochondrial health influences Paneth cell health. ISC niche abnormalities including Paneth cell defects were evident before severe inflammation in TNF^ΔARE^ mice and in uninvolved tissue of CD patients [20]. This suggests that mitochondrial alterations likely comprise early molecular events before the onset of severe disease pathology and that Paneth cells are highly susceptible to mitochondrial dysfunction. Mitochondrial health may be especially important in Paneth cells since they are terminally differentiated, long-lived cells (30–60 days), in which damage mitochondria are not diluted by cell replication [2]. In comparison, enterocytes and goblet cells turnover every 3–5 days, thereby preventing the persistence of damaged mitochondria. Here, stratification of RNAseq results based on Paneth cell phenotyping revealed innate immune response, lipid and protein metabolism, and GPCR signaling as altered by Mito-Tempo in Type I CD patients. Mitochondrial signals are known activators of innate immune signaling including activation of the NLRP3 inflammasome [53]. The downregulation of *SIGLEC14* by Mito-Tempo specifically in Type I CD patients implicates suppressed activation of inflammasome and macrophages [54]. Of note, impaired GPCR signaling is associated with IBD [55]. GPCR signaling was recently shown to be necessary for Paneth cell maturation and mucosal healing, with the absence of Gα_q_/Gα_11_-coupled receptors inducing differentiation of Paneth cells toward goblet cells [56].

Previous studies indicate that Paneth cell defects result from integration of susceptibility gene(s) interaction with environmental triggers [57]. Indeed, a complex interaction of genes, microbiota, and Paneth cell functions are evident given that the Type I Paneth cell phenotype in non-IBD subjects does not correlate with altered mucosal mitochondrial genes or altered gut microbiome [6]. The mechanisms whereby non-IBD patients develop the Type I Paneth cell phenotype are not fully understood. Although the cohort of patients we studied was adult, previous studies demonstrated that both pediatric and adult non-IBD patients exhibit the Type I Paneth cell phenotype [6]. Environmental factors could lead to Paneth cell defects, such as viral infection, which has been shown to cause Paneth cell defects in genetically susceptible mice [18]. Additionally, longitudinal studies are needed to follow these non-IBD patients with the Type I Paneth cell phenotype to determine whether these patients develop IBD, and if so, this would suggest Paneth cell defects might serve as a cellular biomarker for the risk of developing disease [6]. *ATG16L1* T300A, *NOD2*, and *LRRK2* risk alleles were associated with the Type I Paneth cell phenotype in adult CD cohorts depending on ethnic background [3,4,7]. In our cohort of CD patients, the *ATG16L1* T300A risk allele was associated with abnormal Paneth cells. The T300A polymorphism has been shown to reduce autophagy in multiple cell types [57]. In Paneth cells, autophagy has been shown to be important for antimicrobial peptide secretion via secretory autophagy, defense against invading microbes, and resolving ER stress [58]. The present data implicate a role of autophagy in the maintenance of a healthy mitochondrial pool (mitophagy) in Paneth cells. Although mitochondrial health was not reported in all mouse models deficient in autophagy genes resulting in Paneth cell defects, *Atg16l1^T300A^* and *Atg16l^HM^* mice exhibited Paneth cells with degenerating mitochondria [19,59]. Further studies are necessary to determine whether CD susceptibility genes involved in autophagy result in a damaged mitochondrial pool in Paneth cells and the specific role of mitophagy in CD pathogenesis.

The limitations of the present study include the low number of patients recruited into analysis after excluding biopsy samples that had an inadequate number of crypts for Paneth cell phenotyping. Similar exclusion of numerous patients was noted in previous studies using endoscopy biopsies for Paneth cell phenotyping [6]. We focused our patient cohort on adult CD patients, but given that pediatric patients exhibit higher prevalence of Paneth cell defects compared to adults [6], further studies are needed to determine whether CD pediatric patients may especially benefit from mitochondrial-targeted therapy. Although we chose RNA-seq to gain insight into global mucosal pathways altered by a mitochondrial-targeted therapeutic (Mito-Tempo), RNA-seq analysis does not provide transcriptomic analysis for individual cell types present in ileal mucosal biopsies (epithelial cells, immune cells, and fibroblasts/myofibroblasts). To overcome this heterogeneous transcriptomics, single-cell RNA sequencing will be performed in future studies to access alterations within specific epithelial types, such as Paneth cells, with both Type I and Type II Paneth cell phenotype patients included. Our study is limited by the unknown uptake, distribution, and activity of Mito-Tempo within heterogeneous biopsy samples. To gain insight into mitochondrial biogenesis and dynamics during CD, markers in addition to *PPARGC1A* gene expression should be measured, including mitochondrial DNA level and expression of key fission (DRP1, FIS1) and fusion (MFN1/2, OPA1) proteins. Lastly, intestinal biopsies provide a limited snapshot of intestinal physiology. Longitudinal studies will need to be performed to determine whether mitochondrial dysfunction is altered over the course of CD.

These results identify CD enterocytes, goblet cells, and Paneth cells as being susceptible to mitochondrial dysfunction during active inflammation. Type I CD patients with Paneth cell defects are susceptible to mitochondrial dysfunction independent of active inflammation. Protective gene signatures are induced by Mito-Tempo in active CD biopsies. Mitochondrial-targeted therapies may improve outcomes in ileal CD patients and may provide adjuvant therapy for Type I patients to extend remission.

## Figures and Tables

**Table 1:** Characteristics of patients recruited into analysis.

Characteristic	Non-IBD Control	CD	*p*-Value CD vs. Non-IBD
Number of patients	25	30	
Gender, *n* (%)			
Male	11 (44)	19 (63)	0.11
Female	14 (56)	11 (37)	
Median age (range)	61 (25–80)	49 (20–71)	0.0002
Race, *n* (%)			
Caucasian	17 (68)	24 (80)	0.36
African American	8 (32)	3 (10)	0.09
Asian	0	1 (3)	0.99
Hispanic	0	2 (6)	0.50
Harvey Bradshaw Index Score	N/A	0–22	
IBD treatment at biopsy, *n* (%)			
5-ASA		5 (17)	
6-MP/AZA/MTX		6 (20)	
Biologics ^a^		17 (56)	
Steroids		6 (20)	
Antibiotics		0	
NSAIDS		4 (13)	
No treatment		5 (17)	
Tobacco use ^b^, *n* (%)			
Smoker	1 (4)	3 (10)	0.99
Non-smoker	17 (68)	26 (87)	0.07
Other conditions, *n* (%)			
Coronary artery disease	2 (8)	1 (3)	0.59
Diabetes mellitus	3 (12)	4 (13)	0.99
Hypertension	10 (40)	9 (30)	0.78
COPD	1 (4)	0	0.45
Congestive heart failure	2 (8)	1 (3)	0.59
Cerebrovascular event	0	3 (10)	0.24
Peripheral vascular dis.	1 (4)	0	0.45
Ankylosing spondylitis	0	4 (13)	0.12
Rheumatoid Arthritis	2 (8)	1 (3)	0.59
Psoriasis	0	1 (3)	0.99
Spondyloarthropathy	0	2 (7)	0.49

^a^ Infliximab, adalimumab, vedolizumab, ustekinimab. ^b^ Missing data from 7 non-IBD and 1 CD patients. CD, Crohn’s disease; 5-ASA, 5-aminosalicylic acid; 6-MP, 6-mercaptopurin; AZA, Azathioprine; MTX, Methotrexate; NSAIDs, nonsteroidal anti-inflammatory drugs; COPD, chronic obstructive pulmonary disease.

**Table 2:** Charcteristics of CD patients classified as Paneth cell abnormal mitochondria phenotype.

Characteristic	Normal Mitochondria	Abnormal Mitochondria	*p*-Value Normal vs. Abnormal Mitochondria
Number of patients	19	11	
Gender, n (%)			
Male	14 (74)	6 (54)	0.24
Female	5 (26)	5 (46)	
Age, n (%)			
<40	8 (42)	3 (27)	0.47
<50	10 (53)	6 (54)	0.99
<60	14 (74)	8 (72)	0.99
Race, n (%)			
Caucasian	16 (84)	8 (72)	0.64
African American	2 (11)	1 (9)	0.99
Asian	0	1 (9)	0.37
Hispanic	1 (5)	1 (9)	0.99
Harvey Bradshaw Index Score, n (%)			
<5	11 (58)	9 (82)	0.25
5 to 7	5 (26)	2 (18)	0.99
8 to 16	1 (5)	0	0.99
>16	1 (5)	0	0.99
Endoscopically active disease present, n (%)	11 (58)	5 (46)	0.71
IBD treatment at biopsy, n (%)			
5-ASA	3 (16)	2 (18)	0.99
6MP/AZA/MTX	4 (21)	2 (18)	0.99
Biologics	10 (53)	7 (64)	0.71
Steroids	2 (11)	4 (36)	0.16
NSAIDS	1 (5)	3 (27)	0.13
No treatment	3 (16)	2 (18)	0.99
Smoking, n (%)	1 (5)	2 (18)	0.54
Type I Paneth cell phenotype, n (%)	1 (5)	9 (82)	<0.0001
Other conditions, n (%) ^a^			
Diabetes mellitus	1 (5)	3 (27)	0.13
Hypertension	4 (21)	5 (46)	0.23
Cerebrovascular event	1 (5)	2 (18)	0.54
Ankylosing spondylitis	2 (11)	2 (18)	0.61

^a^ Too few patients for analysis for conditions not listed.

## Data Availability

The high-throughput sequencing data from Novogene for this study have been submitted to the NCBI Sequence Read Archive (SRA) under accession number GSE159751.

## Supplementary Materials

The following are available online at https://www.mdpi.com/article/10.3390/cells10061349/s1, Figure S1: Significantly enriched gene signatures in CD vs. non-IBD patients by Reactome analysis; Figure S2: RNA expression of several transcripts in human ileal mucosal biopsies by quantitative real time-PCR; Table S2: The 253 of the 1232 upregulated genes in CD patients restored to non-IBD expression level by Mito-Tempo; Table S3: The 325 of the 1065 downregulated genes in CD patients restored to non-IBD gene expression level by Mito-Tempo; Table S4: The 376 altered genes with significant *p* value; and Table S5: Genes present in Figure 5A Clusters A and D.
