# Supplementary material for: Targeting Mitochondrial Damage as a Therapeutic for Ileal Crohn’s Disease
Source: Cells. 2021 May 29;10(6):1349. doi: 10.3390/cells10061349 (PMC8226558; doi:10.3390/cells10061349)
Supplement: Supplementary file 1 [file cells-10-01349-s001.zip › Figures S1 and S2.pdf]

Supplemental Figure S1

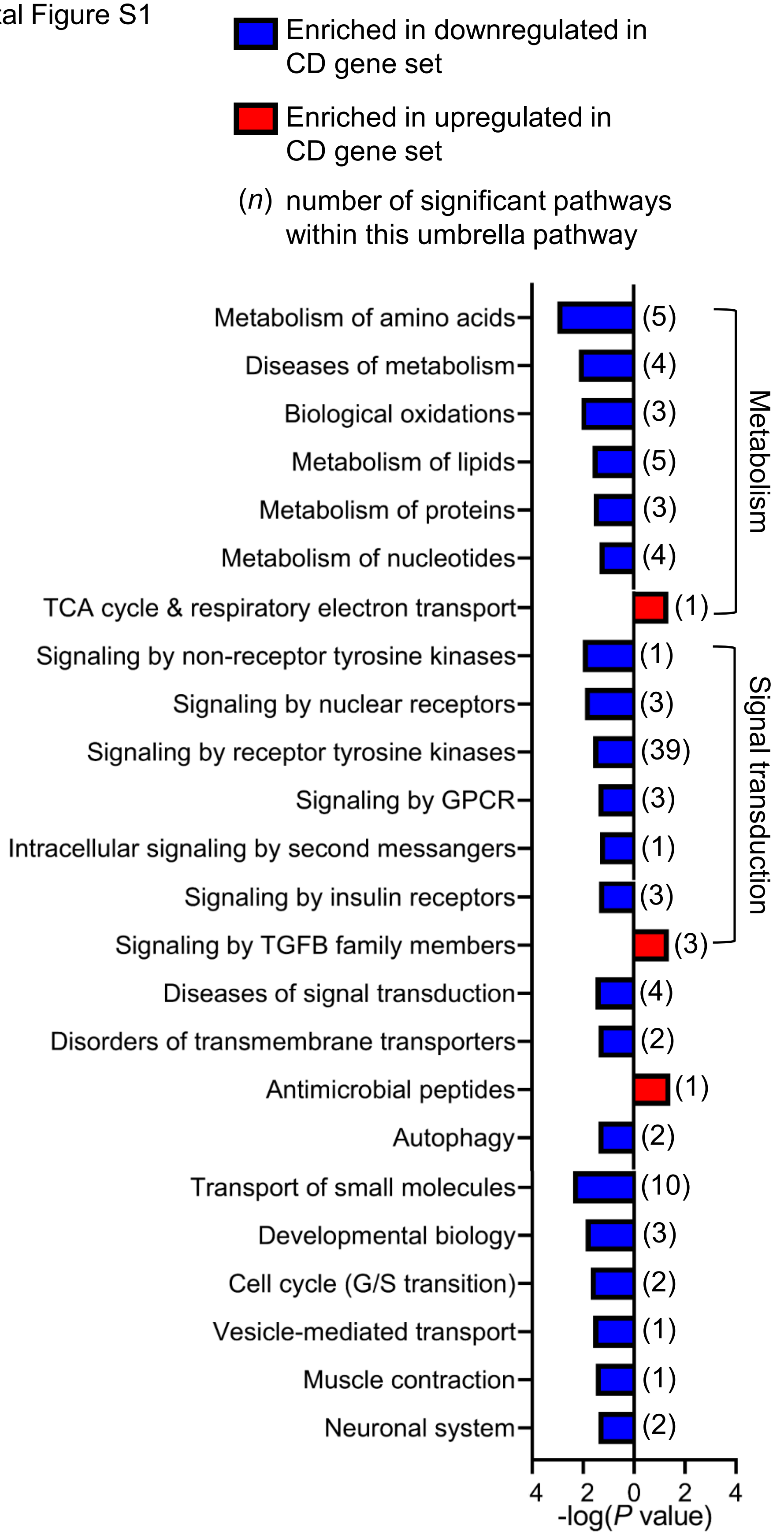

Supplemental Figure S1. Significantly enriched gene signatures in CD vs non-IBD patients by Reactome analysis. General umbrella pathways are indicated by brackets. $P < 0.05$ was considered significant in pathway selection.

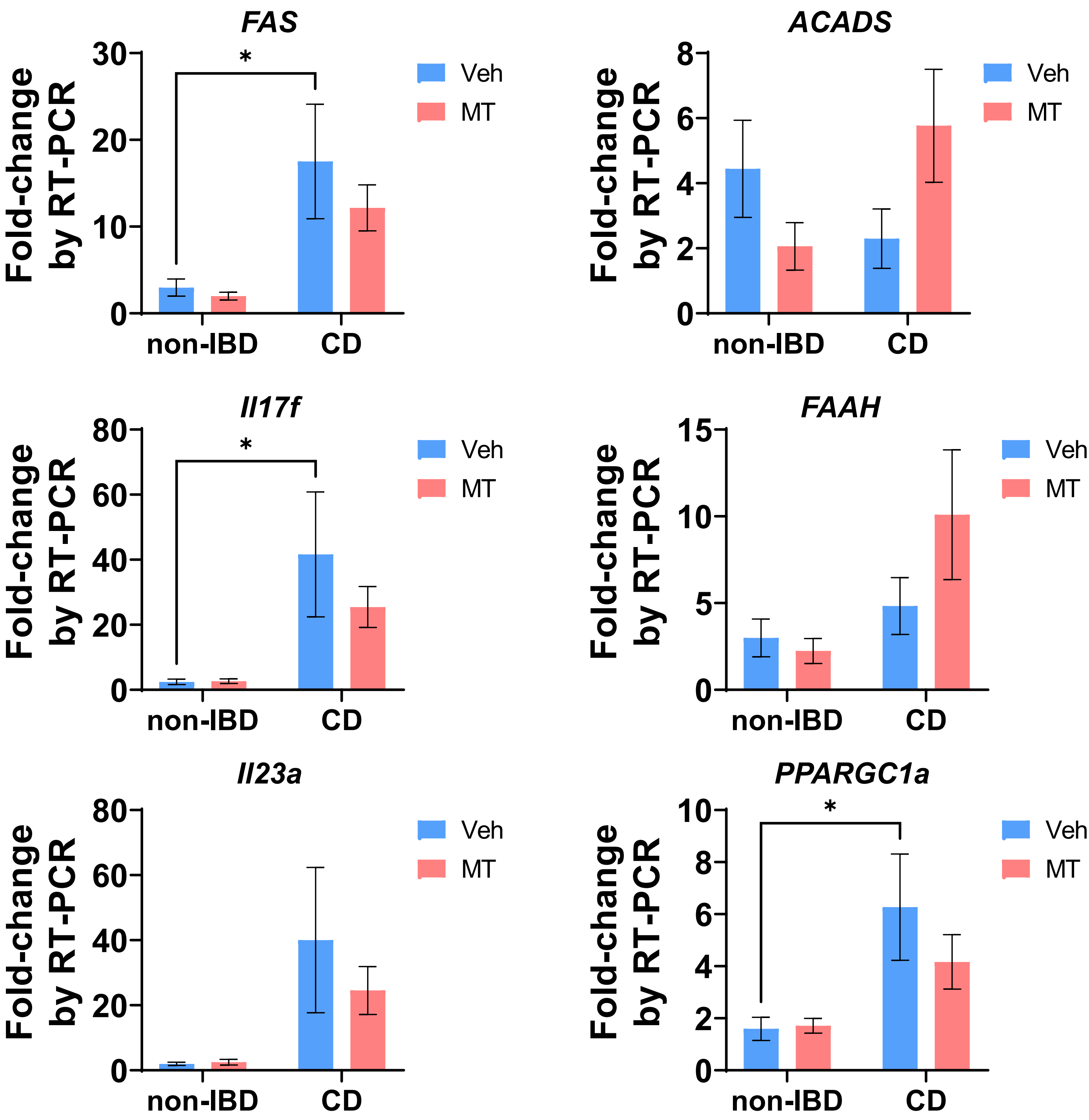

Supplemental Figure S2. RNA expression of several transcripts in human ileal mucosal biopsies by quantitative real time-PCR. Results are presented as mean ± SEM of 9-10 non-IBD veh, 10-12 non-IBD MT, 8-12 CD veh, and 12-14 CD MT biopsies. $^*P < 0.05$ by two-way ANOVA and Tukey's post-hoc test.
